# Association between Flow-Mediated Dilation and Skin Perfusion Pressure with Peripheral Artery Disease in Hemodialysis Patients

**DOI:** 10.3390/jpm11121251

**Published:** 2021-11-25

**Authors:** Chih-Hsuan Wung, Yu-Hsiu Wang, Yuang-Chi Lee, Chieh-Wei Chang, Pei-Yu Wu, Jiun-Chi Huang, Yi-Chun Tsai, Szu-Chia Chen, Jer-Ming Chang, Shang-Jyh Hwang

**Affiliations:** 1 Department of Post Baccalaureate Medicine, Kaohsiung Medical University, Kaohsiung 807, Taiwan; knash031130@hotmail.com.tw; 2 Department of Nursing, Kaohsiung Municipal Siaogang Hospital, Kaohsiung Medical University, Kaohsiung 812, Taiwan; eightshiou@gmail.com (Y.-H.W.); yaungchi2021@gmail.com (Y.-C.L.); stacy97761014@gmail.com (C.-W.C.); 3 Division of Nephrology, Department of Internal Medicine, Kaohsiung Medical University Hospital, Kaohsiung Medical University, Kaohsiung 807, Taiwan; wpuw17@gmail.com (P.-Y.W.); karajan77@gmail.com (J.-C.H.); 4 Department of Internal Medicine, Kaohsiung Municipal Siaogang Hospital, Kaohsiung Medical University, Kaohsiung 812, Taiwan; lidam65@yahoo.com.tw (Y.-C.T.); jemich@kmu.edu.tw (J.-M.C.); 5 Faculty of Medicine, College of Medicine, Kaohsiung Medical University, Kaohsiung 807, Taiwan

**Keywords:** flow-mediated dilation, skin perfusion pressure, ankle-brachial index, brachial-ankle pulse wave velocity, hemodialysis

## Abstract

Flow-mediated dilation (FMD) is used to noninvasively assess the health of blood vessels and it has been shown to have a similar predictive ability for cardiovascular disease to traditional risk factors. Skin perfusion pressure (SPP) refers to the blood pressure required to restore capillary or microcirculatory flow after controlled occlusion and the return of flow. SPP has been shown to be an important measurement when making clinical decisions for patients with limb ischemia and to be a predictor of the likelihood of wound healing. Peripheral artery disease is common in hemodialysis (HD) patients. However, little is known about the association between FMD or SPP and peripheral artery disease. The aim of this study was to evaluate the association between FMD and SPP with brachial-ankle pulse wave velocity (baPWV) and ankle-brachial index (ABI) in HD patients in Taiwan, an area with a high rate of ESRD. This study was conducted at a regional hospital in southern Taiwan. ABI and baPWV values were measured using an ABI automated device. FMD and SPP were measured using ultrasound and a microvasculature blood flow monitor, respectively. Eighty patients were enrolled in this study. Compared to the patients with an ABI ≥ 0.95, those with an ABI < 0.95 had lower SPP of the feet (dorsal and plantar portions, both *p* < 0.001). After multivariable adjustments, low triglycerides (*p* = 0.033) and high calcium–phosphate product (*p* = 0.018) were significantly associated with low FMD. Further, low ABI (*p* = 0.001) and low baPWV (*p* = 0.036) were significantly associated with low SPP of dorsal portions. Old age (*p* = 0.005), low high-density lipoprotein cholesterol (*p* = 0.016), and low ABI (*p* = 0.002) were significantly associated with low SPP of plantar portions. This study demonstrated an association between FMD and SPP with peripheral artery disease in HD patients. Patients with low ABI and baPWV had a high risk of low SPP of the feet. However, there was no significant correlation between FMD and ABI or baPWV.

## 1. Introduction

Cardiovascular (CV) morbidity and mortality rates in hemodialysis (HD) patients are high due to endothelial dysfunction caused by uremic toxins and oxidative stress [1]. Endothelial dysfunction can lead to peripheral artery disease (PAD) [2], which is a common complication in HD patients with rates ranging from 17% to 48% [3,4]. The serious complications of PAD are detrimental to the outcomes of HD patients [5], because PAD has been associated with coronary artery disease, congestive heart failure and foot lesions in many cohort studies [6].

Flow-mediated dilation (FMD) is used to noninvasively assess the health of blood vessels (endothelial dysfunction) and is measured using high-resolution ultrasound [7]. It is expressed as percentage change of the arterial diameter from the baseline diameter [8]. FMD has been used in both clinical research and clinical practice [9], and it has been shown to be strongly predictive of future CV events in patients with diabetes mellitus (DM) [10]. Moreover, two previous studies have demonstrated impaired FMD in patients with end-stage renal disease [11,12]. Skin perfusion pressure (SPP) refers to the blood pressure (BP) required to restore capillary or microcirculatory flow after controlled occlusion and the return of flow [13]. It has been shown to be an important measurement when making clinical decisions for patients with limb ischemia and to be a predictor of the likelihood of wound healing [14]. The advantages of SPP include its non-invasiveness, high reproducibility and independence from the impact of calcification [15].

The ankle-brachial index (ABI) and brachial-ankle pulse wave velocity (baPWV) have been shown to be good markers of PAD and vascular damage and they have both been used to identify PAD in HD patients [16,17,18]. However, little is known about the association between FMD and SPP with PAD. Therefore, the aim of this study was to evaluate the association between FMD, SPP, ABI, and baPWV in Taiwanese HD patients.

## 2. Subjects and Methods

### 2.1. Study Patients and Design

**Figure 1:**
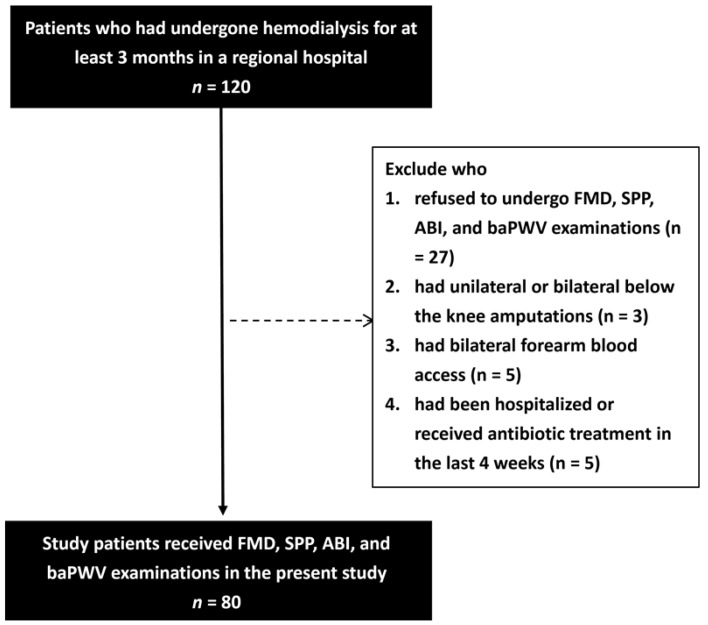
Flowchart of study population.

A study was conducted in the dialysis clinic of a regional hospital in Taiwan in April 2021. The inclusion criteria were patients who had undergone HD for at least three months at age older than 18 years (*n* = 120). The exclusion criteria were patients who: (1) refused to undergo FMD, SPP, ABI, or baPWV examinations (*n* = 27); (2) had unilateral or bilateral below the knee amputations (*n* = 3); (3) had bilateral forearm blood access (*n* = 5); and (4) had been hospitalized or received antibiotic treatment in the last four weeks (*n* = 5) (Figure 1). The remaining 80 patients (47 males and 33 females; mean age 63.8 ± 11.1 years) were included. Each HD session lasted for 3.5–4.0 h, and each patient underwent three sessions per week. The blood flow rate was set at 250–300 mL/min, with a dialysate flow rate of 500 mL/min. The values of FMD, SPP, ABI and baPWV were measured midway through an HD session.

All of the patients provided written informed consent to participate in this study, which was approved by the Institutional Review Board of Kaohsiung Medical University Hospital (KMUHIRB-E(II)-20200315). The methods were carried out in accordance with the approved guidelines.

### 2.2. Assessment of FMD

All studies were performed in a quiet, dark, air-conditioned room (constant temperature of 22–25 °C). The subjects remained supine throughout the study. The vascular response to reactive hyperemia in the brachial artery was assessed for ultrasound assessment of endothelium-dependent FMD [19]. A high-resolution linear artery transducer was coupled to computer-assisted analysis software (UNEXEF18G, UNEX Co, Nagoya, Japan) that used an automated edge detection system for measurement of brachial artery diameter. A blood pressure cuff was placed around the side of the forearm without vascular access. The brachial artery was scanned longitudinally 5 to 10 cm above the elbow. When the clearest B-mode image of the anterior and posterior intimal interfaces between the lumen and vessel wall was obtained, the transducer was held at the same point throughout the scan by a special probe holder (UNEX Co) to ensure the consistency of the image. Depth and gain settings were set to optimize the images of the arterial lumen wall interface. When the tracking gate was placed on the intima, the artery diameter was automatically tracked and the waveform of diameter changes over the cardiac cycle was displayed in real time with the use of the FMD mode of the tracking system. This allowed the ultrasound images to be optimized at the start of the scan and the transducer position to be adjusted immediately for optimal tracking performance throughout the scan. Pulsed Doppler flow was assessed at baseline and during peak hyperemic flow, which was confirmed to occur within 15 s after cuff deflation. Blood flow velocity was calculated from the color Doppler data and was displayed as a waveform in real time. The baseline longitudinal image of the artery was acquired for 30 s, and then the blood pressure cuff was inflated to 50 mm Hg above systolic pressure for 5 min. The longitudinal image of the artery was recorded continuously until 5 min after cuff deflation. Pulsed Doppler velocity signals were obtained for 20 s at baseline and for 10 s immediately after cuff deflation. Changes in brachial artery diameter were immediately expressed as percent change relative to the vessel diameter before cuff inflation. FMD was automatically calculated as the percent change in peak vessel diameter from the baseline value. The measurement %FMD (peak diameter−baseline diameter/baseline diameter) was used for analysis. Blood flow volume was calculated by multiplying the Doppler flow velocity (corrected for the angle) by heart rate and vessel cross-sectional area (πr2). Reactive hyperemia was calculated as the maximum percent increase in flow after cuff deflation compared with baseline flow. The FMD measurements were done once in each patient.

### 2.3. Assessment of SPP of the Feet

SPP measurements of the plantar and dorsal portions of the feet were obtained in each patient using a microvasculature blood flow monitor (Nahri MV monitor; Nexis, Tokyo, Japan). Suprasystolic compression was performed at the measurement point to stop blood flow and then the cuff was slowly deflated until the return of blood flow was detected by an optical sensor utilizing laser Doppler technology. Examinations were usually performed in the afternoon after the patients had been allowed to rest for around 30 min. If the patient had an open wound at the measurement site, SPP was measured as close to the wound as possible. Both feet of each patient were measured twice, and the four times were averaged.

### 2.4. Assessment of ABI and baPWV

The values of ABI and baPWV were measured using an ABI automated device (VP1000; Colin Co. Ltd., Komaki, Japan), which automatically and simultaneously measures blood pressures in both arms and ankles using an oscillometric method [20]. Monitoring and occlusion cuffs were attached firmly around both sides of the lower extremities and the upper arm (without vascular access) of the patient while they were in the supine position. ABI was calculated as the lower value of ankle systolic BP divided by that in the arm. ABI was measured once in each patient. The lower of bilateral ABI values was used in the analysis. Patients with PAD were defined as those with ABI < 0.95 [21].

BaPWV was measured using an automated oscillometric device that recorded pulse waves in the brachial and posterior tibial arteries [22,23,24]. The transmission time (ΔTba) was calculated as the time between the initial increases in brachial and ankle waveforms. The brachial to ankle transmission distance was then calculated based on the height of the patient. The distances from the suprasternal notch to the brachium (Lb) and ankle (La) were calculated as: Lb = 0.2195 × height of the patient (in cm) − 2.0734; and La = 0.8129 × height of the patient (in cm) + 12.328. BaPWV was then calculated as (La − Lb)/ΔTba. The higher of the bilateral baPWV values was used in the analysis. BaPWV was measured once in each patient.

### 2.5. Collection of Demographic, Medical, and Laboratory Data

The medical records of each patient were used to obtain data on age, sex and a history of diabetes, hypertension, coronary artery disease, and cerebrovascular disease. Blood samples were obtained after a 12 h fast within 1 month of enrollment and analyzed using an autoanalyzer (COBAS Integra 400, Roche Diagnostics GmbH, D-68298 Mannheim, Germany). All of the participants also underwent physical examinations, during which body weight and height were recorded. Body mass index (BMI) was calculated as kg/m^2^.

### 2.6. Statistical Analysis

Descriptive statistics are presented as number (percentage) or mean ± standard deviation. Between-group comparisons were performed using the independent *t* test for continuous variables and chi-square test for categorical variables. Multivariable stepwise linear regression analysis was used to evaluate the association between ABI and baPWV with FMD and SPP. A *p* value < 0.05 was considered to be statistically significant. All statistical analyses were performed using SPSS version 22.0 for Windows (SPSS Inc., Chicago, IL, USA).

## 3. Results

### 3.1. Comparison of the Clinical Characteristics among the Patients with an ABI < 0.95 or ≥ 0.95

The characteristics of the patients with an ABI < 0.95 or ≥ 0.95 are shown in Table 1. Compared to the patients with an ABI ≥ 0.95, those with an ABI < 0.95 had higher prevalence rates of diabetes and cerebrovascular disease, lower diastolic BP, higher BMI, lower high-density lipoprotein (HDL)-cholesterol, and lower SPP of the feet (dorsal and plantar portions, both *p* < 0.001).

### 3.2. Risk Factors for FMD

The factors associated with FMD in multivariable stepwise linear regression analysis after adjusting for age, sex, DM, hypertension, coronary artery disease, cerebrovascular disease, BMI, fasting glucose, albumin, hemoglobin, triglycerides, total cholesterol, HDL-cholesterol, low-density lipoprotein cholesterol, calcium–phosphate product, ABI and baPWV are shown in Table 2. Low triglycerides (per 1 mg/dL; unstandardized coefficient β, 0.007; 95% confidence interval [CI], 0.001 to 0.013; *p* = 0.033) and high calcium–phosphate product (per 1 mg^2^/dL^2^; unstandardized coefficient β, −0.084; 95% CI, −0.152 to −0.015; *p* = 0.018) were significantly associated with low FMD. However, ABI and baPVW did not achieve significance.

Multivariable adjusted for age, sex, DM, hypertension, coronary artery disease, cerebrovascular disease, BMI, fasting glucose, albumin, hemoglobin, triglyceride, total cholesterol, HDL-cholesterol, LDL-cholesterol, calcium–phosphate product, ABI and baPWV.

We further performed a subgroup analysis after the exclusion of two patients with ABI > 1.3, and found similar results. Low triglycerides and high calcium–phosphate product were significantly associated with low FMD.

### 3.3. Risk Factors for SPP of Dorsal Portions

The factors associated with SPP of dorsal portions are shown in Table 3. After multivariable adjustments, low ABI (per 0.1; unstandardized coefficient β, 6.561; 95% CI, 2.719 to 10.402; *p* = 0.001) and low baPWV (per 100 cm/s; unstandardized coefficient β, 1.442; 95% CI, 0.101 to 2.783; *p* = 0.036) were significantly associated with low SPP of dorsal portions.

Multivariable was adjusted for age, sex, DM, hypertension, coronary artery disease, cerebrovascular disease, BMI, fasting glucose, albumin, hemoglobin, triglyceride, total cholesterol, HDL-cholesterol, LDL-cholesterol, calcium–phosphate product, ABI and baPWV.

We further performed a subgroup analysis after the exclusion of two patients with ABI > 1.3 and found similar results. Low ABI and low baPWV were significantly associated with low SPP of dorsal portions.

### 3.4. Risk Factors for SPP of Plantar Portions

The factors associated with SPP of plantar portions are shown in Table 4. After multivariable adjustments, old age (per 1 year; unstandardized coefficient β, −0.958; 95% CI, −1.612 to −0.304; *p* = 0.005), low HDL-cholesterol (per 1 mg/dL; unstandardized coefficient β, 0.722; 95% CI, 0.144 to 1.300; *p* = 0.016), and low ABI (per 0.1; unstandardized coefficient β, 5.289; 95% CI, 2.091 to 8.486; *p* = 0.002) were significantly associated with low SPP of plantar portions.

Multivariable was adjusted for age, sex, DM, hypertension, coronary artery disease, cerebrovascular disease, BMI, fasting glucose, albumin, hemoglobin, triglyceride, total cholesterol, HDL-cholesterol, LDL-cholesterol, calcium–phosphate product, ABI and baPWV.

We further performed a subgroup analysis after the exclusion of two patients with ABI > 1.3 and found the similar results. Old age, low HDL-cholesterol, and low ABI were significantly associated with low SPP of plantar portions.

## 4. Discussion

In this study, we evaluated the association between FMD and SPP with ABI and baPWV in 80 HD patients. We found that a low ABI and low baPWV were associated with low SPP of dorsal portions and that a low ABI was associated with low SPP of plantar portions. However, there was no significant correlation between FMD with ABI and baPWV.

The first finding of our study is that there was no correlation between FMD and ABI or baPWV. Gupta et al. reported a positive correlation between FMD and ABI in young males post myocardial infarction in India [25]. However, Vigna et al. found no significant correlation between ABI and FMD in a dyslipidemic population [26], and Kaczmarczyk et al. reported no correlation between ABI, toe-brachial index and FMD among patients with PAD [27]. Taken together with our finding of no correlation between FMD and ABI among HD patients, it appears that the relationship between FMD and ABI varies among different populations. A possible explanation of no correlation between FMD and ABI or baPWV is that FMD is predominantly affected by nitrogen oxide (NO) [8], whereas PAD develops through lower limb artery atherosclerosis, an imbalance in lipid metabolism and chronic inflammation. On the other hand, Tomiyama et al. reported a non-significant correlation between FMD and baPWV among hypertensive patients [28], and Nakaruma et al. also reported no significant difference between FMD and baPWV among patients with coronary artery disease [29]. We also found no correlation between FMD and baPWV among HD patients. A reasonable explanation is that FMD mainly reflects endothelial dysfunction [30], whereas baPWV reflects arterial stiffening through both structural and functional changes including endothelial dysfunction, elevated smooth muscle tone and medial hypertrophy related to the development of atherosclerosis [31]. Vascular endothelial dysfunction is the first step of atherosclerosis, [32], and the progression of atherosclerosis may partly explain why there was no significant correlation between FMD with ABI and baPWV in this study.

The second finding of this study is that low ABI was associated with low SPP of both dorsal and plantar portions of the feet and that low baPWV was associated with low SPP of dorsal portions. Davis et al. and Ishioka et al. also found that SPP was positively associated with ABI among HD patients [33,34] and Shimazaki et al. found that SPP decreased significantly in HD patients with lower ABI compared to those with normal ABI [35]. SPP reflects blood flow in the microcirculatory system, hence it can be used to assist in the assessment of PAD [36]. Marginal ischemia with limited wound healing in PAD patients has been associated with a low SPP value [14]. In this study, we also found that low baPWV was associated with low SPP of dorsal portions. A possible explanation is vessel occlusion. Obvious stenosis or occlusion of blood vessels would affect the velocity of pulse waves, leading to lower baPWV values [37]. At the same time, occlusion would affect the restoration of microcirculatory or capillary flow, leading to a lower SPP value [13]. However, further studies are needed to elucidate this hypothesis.

The third finding of this study is that a low triglyceride level was significantly associated with low FMD. Samsamshariat et al. found no association between FMD and triglycerides among both subjects with and without metabolic syndrome [38]. In addition, Fukumoto et al. found that FMD was inversely correlated with triglyceride level among patients with mild hypertriglyceridemia [39], and Kajikawa et al. demonstrated that higher serum triglyceride levels were associated with lower FMD. However, we found that a low triglyceride level was associated with low FMD among HD patients. Increased serum levels of triglycerides have been associated with endothelial dysfunction in several populations, including those with metabolic syndrome, chronic kidney disease and coronary artery disease [40,41,42]. Increasing evidence suggests that malnutrition and inflammation are common in HD patients [43], in whom a low triglyceride level is considered to be an indicator of malnutrition. Malnutrition, inflammation and atherosclerosis interact with inflammatory cytokines and tumor necrosis factor-α, eventually leading to endothelial dysfunction [44]. Nevertheless, further studies are needed to confirm the association between triglyceride level and FMD among different populations.

Another interesting finding of this study is that high calcium–phosphate product was significantly associated with low FMD. Hyperphosphatemia can increase calcium–phosphate production through direct and indirect mechanisms [45], and both hyperphosphatemia and calcium–phosphate product have been shown to play a key role in vascular calcification in HD patients [46]. Stevens et al. reported that phosphate supplements were associated with a significant reduction in FMD among healthy volunteers [47]. A possible molecular mechanism is that hyperphosphatemia alters the vascular endothelial growth factor signaling pathway and increases p53 expression, leading to a reduction in total phosphate and endothelial NO synthase expression. Eventually, this reduction in NO results in endothelial dysfunction [47].

The last finding is that low HDL-cholesterol was associated with low SPP of plantar portions. HDL-cholesterol is a complex molecule with many potentially atheroprotective activities such as reverse cholesterol transport, inhibiting the expression of adhesion molecules on endothelial cells and stimulating endothelial NO synthase to promote vasodilation [48]. A possible explanation for our finding is that low HDL-cholesterol has been shown to decrease the activation of endothelial NO synthase, leading to impaired vasodilation [49]. Impaired restoration of vasodilation would then lead to a decrease in the SPP value.

There are several limitations to this study. First, only 80 patients were enrolled, so studies with a larger number of participants are needed to confirm our findings. The limited number of study patients severely reduced the power of the study. Second, as all of the study patients were enrolled from a single regional hospital in Taiwan, our findings may not be generalizable to other areas. Third, due to the cross-sectional study design, we were unable to evaluate causal relationships and long-term clinical outcomes. Fourth, the sensitivity of ABI and baPWV for detecting PAD in HD patients has been reported to be low [50]. Nonetheless, our results highlight the importance of FMD and SPP on PAD in HD patients.

In conclusion, we identified an association between SPP of the feet with ABI and baPWV. However, there was no correlation between FMD and ABI or baPWV in the HD patients in this study.

## Figures and Tables

**Table 1:** Comparison of clinical characteristics among HD patients with ABI < 0.95 or ≥ 0.95.

Characteristics	ABI ≥ 0.95 (*n* = 51)	ABI < 0.95 (*n* = 29)	*p*
Age (year)	62.6 ± 12.3	65.6 ± 8.2	0.246
Male number (%)	30 (58.8)	17 (58.6)	0.986
DM number (%)	22 (43.1)	21 (72.4)	0.012
Hypertension number (%)	36 (70.6)	23 (79.3)	0.394
Coronary artery disease number (%)	11 (21.6)	11 (37.9)	0.115
Cerebrovascular disease number (%)	5 (9.8)	9 (31.0)	0.013
Systolic blood pressure (mmHg)	138.5 ± 22.1	128.7 ± 26.8	0.083
Diastolic blood pressure (mmHg)	77.6 ± 14.2	70.8 ± 9.2	0.022
Pulse pressure (mmHg)	60.9 ± 12.3	58.0 ± 20.0	0.477
BMI (kg/m^2^)	23.4 ± 4.1	25.5 ± 3.7	0.027
Laboratory parameters			
Fasting glucose (mg/dL)	148.1 ± 58.7	135.2 ± 51.7	0.459
Albumin (g/dL)	3.9 ± 0.2	3.9 ± 0.2	0.859
Hemoglobin (g/dL)	10.8 ± 0.8	11.1 ± 1.0	0.151
Triglyceride (mg/dL)	128.8 ± 95.8	129.6 ± 69.5	0.971
Total cholesterol (mg/dL)	157.4 ± 30.3	153.1 ± 40.8	0.600
HDL-cholesterol (mg/dL)	44.9 ± 16.1	37.5 ± 8.8	0.010
LDL-cholesterol (mg/dL)	77.3 ± 26.2	81.6 ± 31.8	0.524
Calcium–phosphate product (mg^2^/dL^2^)	42.2 ± 10.1	45.0 ± 11.9	0.284
Exams			
ABI	1.1 ± 0.1	0.8 ± 0.1	<0.001
baPWV (cm/s)	1951. 8 ± 395.8	1828.3 ± 557.9	0.253
FMD (%)	3.6 ± 2.5	3.4 ± 2.0	0.711
SPP of dorsal portion of foot (mmHg)	103.4 ± 20.9	82.0 ± 23.9	<0.001
SPP of plantar portion of foot (mmHg)	88.2 ± 21.7	64.0 ± 21.6	<0.001

Abbreviations. HD, hemodialysis; ABI, ankle-brachial index; baPWV, brachial-ankle pulse wave velocity; DM, diabetes mellitus; BMI, body mass index; HDL, high-density lipoprotein; LDL, low-density lipoprotein; FMD, flow-mediated dilation; SPP, skin perfusion pressure.

**Table 2:** Determinants for FMD using multivariable stepwise linear regression analysis.

Characteristics	Multivariable (Stepwise)	
	Unstandardized Coefficient β (95% CI)	*p*
Triglyceride (per 1 mg/dL)	0.007 (0.001, 0.013)	0.033
calcium–phosphate product (per 1 mg^2^/dL^2^)	−0.084 (−0.152, −0.015)	0.018

Values expressed as unstandardized coefficient β and 95% confidence interval (CI). Abbreviations are the same as in Table 1.

**Table 3:** Determinants for SPP of dorsal portion of foot using multivariable stepwise linear regression analysis.

Characteristics	Multivariable (Stepwise)	
	Unstandardized Coefficient β (95% CI)	*p*
ABI (per 0.1)	6.561 (2.719, 10.402)	0.001
baPWV (per 100 cm/s)	1.442 (0.101, 2.783)	0.036

Values expressed as unstandardized coefficient β and 95% confidence interval (CI). Abbreviations are the same as in Table 1.

**Table 4:** Determinants for SPP of plantar portion of foot using multivariable stepwise linear regression analysis.

Characteristics	Multivariable (Stepwise)	
	Unstandardized Coefficient β (95% CI)	*p*
Age (per 1 year)	−0.958 (−1.612, −0.304)	0.005
HDL-cholesterol (per 1 mg/dL)	0.722 (0.144, 1.300)	0.016
ABI (per 0.1)	5.289 (2.091, 8.486)	0.002

Values expressed as unstandardized coefficient β and 95% confidence interval (CI). Abbreviations are the same as in Table 1.

## Data Availability

Data may be available upon request to interested researchers. Please send data requests to: Szu-Chia Chen, PhD, MD. Division of Nephrology, Department of Internal Medicine, Kaohsiung Medical University Hospital, Kaohsiung Medical University.
